# Low pregnancy-associated plasma protein-A and serum urotensin-II in 11–14 weeks gestation: case-control study

**DOI:** 10.1590/1806-9282.20260478

**Published:** 2026-08-28

**Authors:** Yusa Irfan Yildirim, Rojda Bayar Yildirim, Murat Muhcu

**Affiliations:** 1 Antalya Kepez State Hospital, Department of Obstetrics and Gynecology – Antalya, Türkiye.; 2 Antalya Kepez State Hospital, Department of Delivery Room – Antalya, Türkiye.; 3 Hamidiye University of Health Sciences, Department of Obstetrics and Gynecology, Faculty of Medicine – Istanbul, Türkiye.

**Keywords:** Pregnancy, first trimester, Pregnancy-associated plasma protein-A, Biomarkers, Prenatal diagnosis, Case-control studies

## Abstract

**OBJECTIVE:**

This case-control study aims to investigate the relationship between low pregnancy-associated plasma protein-A levels and serum urotensin-II concentrations between the 11th and 14th weeks of pregnancy.

**METHODS:**

This study was conducted between October 2024 and October 2025 at the perinatology outpatient clinic of a Training and Research Hospital in Istanbul. A total of 84 women were consecutively included and divided into case and control groups based on pregnancy-associated plasma protein-A results. Written consent and a descriptive form were obtained. Combined screening tests were performed, and blood samples were collected for urotensin-II measurement.

**RESULTS:**

No significant difference was observed between the case and control groups in terms of urotensin-II concentration. A statistically significant positive correlation was found between pregnancy-associated plasma protein-A levels and urotensin-II concentrations. Each 1 mIU/mL increase in pregnancy-associated plasma protein-A concentration was associated with an approximate 36.45 ng/mL increase in urotensin-II concentration.

**CONCLUSION:**

In this study, no significant association was demonstrated between pregnancy-associated plasma protein-A levels and urotensin-II levels in case and control groups; however, a correlation was identified between pregnancy-associated plasma protein-A levels and urotensin-II concentrations.

## INTRODUCTION

Pregnancy-associated plasma protein-A (PAPP-A) is a metalloproteinase synthesized by the syncytiotrophoblasts and decidua that cleaves insulin-like growth factor-binding proteins (IGFBPs). Through this mechanism, it increases the ­bioavailability of insulin-like growth factors (IGF), thereby regulating trophoblast invasion, placental development, and fetoplacental growth^1^. Low PAPP-A levels have been reported to contribute to placental insufficiency by reducing IGF bioavailability^2^. In addition, PAPP-A is a key biochemical marker used in the combined first-trimester screening test^3^. The combined test is a screening tool performed between 11 and 14 weeks of gestation to assess the risk of chromosomal abnormalities. It incorporates measurements of nuchal translucency (NT), crown–rump length (CRL), maternal age, free beta-human chorionic gonadotropin (β-hCG), and PAPP-A expressed as multiples of the median (MoM)^4,5^. Urotensin-II (UT-II) is recognized as one of the most potent endogenous vasoconstrictor peptides. It is involved in the regulation of vascular tone, smooth muscle cell proliferation, and vascular remodeling in cardiovascular, renal, neurological, and endocrine systems^6^. Additionally, UT-II expression has been identified in placental cytotrophoblast and syncytiotrophoblast cells, suggesting a potential role in placental development and function^7^. Owing to its potential as both a diagnostic biomarker and a pharmacological target, its significance in scientific research has been increasingly recognized^8^. Pregnancy is a physiological period characterized by pronounced hemodynamic adaptations within the uteroplacental circulation^9^. Where this adaptation proves insufficient, placenta-related complications such as pre-­eclampsia and fetal growth restriction (FGR) may arise. It has been suggested that UT-II, due to its potent vasoconstrictor effect and its influence on biological pathways associated with endothelial dysfunction, may be implicated in the vascular ­pathophysiology of pregnancy^10^. Accordingly, reduced IGF bioavailability associated with low PAPP-A levels may impair placental function and alter UT-II expression or signaling. Such alterations may contribute to uteroplacental vasoconstriction, inflammation, pyroptosis, and autophagy, ultimately promoting the development of placental-mediated pregnancy complications, including preeclampsia and FGR. Evidence on the relationship between PAPP-A and UT-II levels in early pregnancy is limited. This study aimed to investigate the association between low PAPP-A levels and serum UT-II concentrations between 11 and 14 weeks of gestation.

## METHODS

### Aim and study design

This case–control study aims to investigate the association between low PAPP-A levels and serum urotensin II concentrations between the 11th and 14th weeks of gestation. The study was conducted in accordance with the STROBE Statement (Strengthening the Reporting of Observational Studies in Epidemiology).

### Hypotheses

H0: There is no association between low PAPP-A levels and serum UT-II levels.

H1: There is an association between low PAPP-A levels and serum UT-II levels.

### Study variables

The independent variables were defined as PAPP-A concentration, PAPP-A MoM, free β-hCG concentration, β-hCG MoM, NT, NT MoM, and CRL measurements. The dependent variable was defined as serum UT-II concentration.

### Study setting and duration

The study was conducted between October 2024 and October 2025 at the Perinatology Clinic of a Training and Research Hospital in Istanbul.

### Population and sample

The study population comprised pregnant women who attended the clinic during the study period. The sample size was calculated using G*Power (v3.1.9); reference values for UT-II levels were taken from the study by Liu et al., comparing control and severe preeclampsia groups with α=0.05, power=0.95, and Cohen’s d=0.729; a total of 84 participants, 42 in each group, were determined^11^.

### Inclusion and exclusion criteria

Inclusion criteria were gestational age between 11 and 14 weeks, completion of the combined screening test, provision of written informed consent, and ability to speak and understand Turkish.

Exclusion criteria were illiteracy, presence of cognitive or hearing impairments, diagnosed psychiatric disorders (e.g., schizophrenia and bipolar disorder), chronic hypertension, and a high-risk result on the combined screening test.

### Ethical approval

The study was conducted with the approval of the Clinical Research Ethics Committee of Istanbul Umraniye Training and Research Hospital (Decision No: 2024/371, Date: October 1, 2024) and was designed in accordance with the Declaration of Helsinki on Human Rights.

### Data collection instruments

Data were initially collected using a Sociodemographic Characteristics Questionnaire and an Informed Consent Form (ICF) prepared by the researchers^10,11^. Subsequently, NT and CRL measurements were obtained via ultrasonography. In addition, venous blood samples were collected to determine PAPP-A, free β-hCG, and UT-II levels, which were measured in serum.

### Study procedure

Eligible pregnant women who attended the perinatology clinic of the public hospital and voluntarily agreed to participate in the study were interviewed individually in person, during which the study objectives and procedures were explained. Participants in both the case and control groups were fully informed prior to the procedure, and written and verbal consents were obtained. A sociodemographic characteristics questionnaire was administered, followed by the combined screening test. Based on the PAPP-A MoM values reported in the screening test, participants were assigned to case and control groups: those with PAPP-A≤0.5 MoM constituted the case group (n=42), while those with PAPP-A>0.5 MoM formed the control group (n=42). Subsequently, serum UT-II levels were measured.

### Serum urotensin-II sampling and analysis

The researcher received instruction and training from a biochemistry specialist affiliated with the relevant company regarding the collection, storage, and transfer of blood serum. A 5 mL venous blood sample was collected from each participant to measure UT-II concentration. Following venous blood collection, samples were allowed to clot at room temperature for 10–20 min and were refrigerated for 2 h at +4°C and then centrifuged at 4,000×*g* for 10 min using a Roche Cobas 6000 device. The serum remaining in the tube after centrifugation was transferred into labeled Eppendorf tubes using an automatic pipette. The samples were subsequently stored at -80°C until analysis in the Biochemistry Laboratory of Health Sciences University Istanbul Umraniye Training and Research Hospital. To preserve biological stability, the cold chain was maintained throughout sample handling, and all samples were processed according to the same storage protocol. On the day of analysis, serum samples were transported to an external private biochemistry laboratory under cold-chain conditions. Upon arrival at the laboratory, the analytical procedure was initiated immediately in accordance with the manufacturer’s protocol. Prior to analysis, samples were thawed in a controlled manner and brought to room temperature. All samples were thawed only once before analysis, and repeated freeze–thaw cycles were avoided to minimize potential effects on analyte stability. Hemolyzed samples were excluded from the study.

UT-II concentrations were measured according to the manufacturer’s instructions using a commercially available enzyme-linked immunosorbent assay (ELISA) kit (Human Urotensin II [UII] ELISA Kit, YLBiont, Cat. No: YLA0797HU, Lot No: YLZIDEU3, expiration date: August 2026) on a BioTek Instruments Inc. ELx800™ device, with measurements processed using Gen8 software. The assay range of the kit was 5–1,000 ng/mL, and the analytical sensitivity (limit of detection [LOD]) was reported as 2.23 ng/mL. The limit of quantification (LOQ) was not specified by the manufacturer.

The calibration curve was generated using serial dilutions of the 1,280 ng/mL stock standard solution provided with the kit. Standard concentrations of 640, 320, 160, 80, and 40 ng/mL were prepared and used to construct the standard curve based on their corresponding optical density values. Sample concentrations were calculated from this calibration curve. Samples with concentrations exceeding the assay range were appropriately diluted and reanalyzed. No sample concentration was below the lower detection limit of the assay.

According to the manufacturer’s specifications, the intra-assay coefficient of variation (CV) was <8% and the inter-assay CV was <10%. For the assessment of intra-assay precision, three samples with low, medium, and high concentrations were analyzed 20 times on the same plate. Inter-assay precision was evaluated by analyzing the same three concentration levels on three different plates with eight replicates per plate. The CV was calculated using the formula CV (%)=SD/mean×100. For quality control purposes, all reagents were stored under the conditions recommended by the manufacturer. Washing concentrate (30×) and stop solution were stored at 4°C, whereas all other reagents were stored at -20°C. Reagents from different lots were not mixed, and all analyses were performed according to the same standardized protocol.

### Statistical analysis

Data were analyzed using SPSS version 20. The internal consistency of the scales was assessed with Cronbach’s alpha, and distribution was examined using the Kolmogorov-Smirnov test. Continuous variables were presented as mean±standard deviation, while categorical variables were reported as frequencies and percentages. Independent-samples t-tests were applied for continuous variables between groups, and the Chi-square test was used for categorical data. Relationships between continuous variables were evaluated using Pearson correlation analysis, and the association between UT-II and PAPP-A was examined through simple linear regression. A significance level of p<0.05 was adopted.

## RESULTS

Laboratory and ultrasonographic measurements of the case and control groups were compared. No significant difference was observed between the groups in terms of UT-II concentration (Table 1). Similarly, no differences were found between the groups for free β-hCG MoM, NT, NT MoM, and CRL measurements (Table 1).

**Table 1:** Comparison of participants’ laboratory results and ultrasonographic measurements.

	Case group (n: 42)	Control group (n: 42)	Mean difference (case—control)	Test Req.	p
	Mean±SD Median (min–max)	Mean±SD Median (min–max)	95%CI	t	
Urotensin-II concentration (ng/mL)	334.37±414.02 158.75 (40.70–1,964.70)	445.47±461.14 221.70 (33.40–1,723.40)	-111.10 (-301.33 to 79.13)	-1.16	0.25
PAPP-A concentration (mIU/mL)	1.31±0.53 1.29 (0.39–3.24)	4.58±3.04 4.12 (0.88–14.90)	-3.27 (-4.22 to -2.32)	-6.856	**0.0003**
PAPP-A MoM	0.41±0.08 0.42 (0.21–0.50)	1.33±0.7 1.14 (0.56–3.50)	-0.92 (-1.14 to -0.71)	-8.530	**0.0004**
β-hCG concentration (ng/mL)	27.27±18.9 23.02 (9.29–121.60)	33.49±19.73 28.59 (12.04–101.50)	-6.22 (-14.61 to 2.17)	-1.476	0.144
β-hCG MoM	0.84±0.68 0.74 (0.32–4.58)	0.97±0.46 0.81 (0.37–2.32)	-0.13 (-0.38 to 0.12)	-1.038	0.303
NT (mm)	1.51±0.42 1.50 (1.00–2.80)	1.6±0.44 1.50 (0.70–3.00)	-0.09 (-0.28 to 0.10)	-0.957	0.341
NT MoM	0.93±0.22 0.88 (0.58–1.49)	0.95±0.19 0.92 (0.52 to 1.53)	-0.02 (-0.11 to 0.07)	-0.515	0.608
CRL (mm)	65.05±9.7 65.50 (50.00–81.00)	65.12±9.9 66.00 (47.00–84.00)	-0.07 (-4.33 to 4.18)	-0.033	0.973

SD: standard deviation; Min–max.: minimum–maximum; CI: confidence interval; t: independent sample T test, n: number of participants; PAPP-A: pregnancy-associated plasma protein-A; MoM: multiple of the median; β-hCG: beta human chorionic gonadotropin; NT: nuchal translucency; CRL: Crown-Rump Length; mIU/mL: milli-international unit per milliliter; ng/mL: nanogram per milliliter; mm: Millimeter; p<0.05. It was considered statistically significant. Bold text indicates statistically significant values (p<0.05).

The participants’ UT-II levels were compared with biochemical and ultrasonographic parameters. UT-II demonstrated a weak but statistically significant positive correlation with PAPP-A concentration (r=0.226, p=0.039) and PAPP-A MoM values (r=0.273, p=0.012). No significant relationships were observed between UT-II and free β-hCG, β-hCG MoM, NT, NT MoM, or CRL (Table 2).

**Table 2:** Investigation of the correlation between participants’ biochemical and ultrasonographic parameters and urotensin-II levels.

		PAPP-A concentration (mIU/mL)	PAPP-A MoM	β-hCG Concentration (ng/mL)	β-hCG MoM	NT (mm)	NT MoM	CRL (mm)
Urotensin-II concentration (ng/mL)	r	0.226	0.273	0.046	0.053	0.056	0.016	0.006
	95%CI	0.012–0.420	0.062–0.460	-0.170 to 0.258	-0.163 to 0.264	-0.160 to 0.267	-0.199 to 0.230	-0.209 to 0.220
	p	**0.039**	**0.012**	0.679	0.632	0.614	0.888	0.955

r: Pearson correlation; CI: confidence interval; PAPP-A: pregnancy-associated plasma protein-A; MoM: multiple of the median; β-hCG: beta human chorionic gonadotropin; NT: nuchal translucency; CRL: crown-rump length; mIU/mL: milli-international unit per milliliter; ng/mL: nanogram per milliliter; mm: millimeter. p<0.05. It was considered statistically significant. Bold text indicates statistically significant values (p<0.05).

The relationship between UT-II levels and PAPP-A concentration was examined using simple linear regression. The model was found to be statistically significant [F(1,82)=4.42; p=0.039]. Each 1 mIU/mL increase in serum PAPP-A concentration was associated with an approximate 36.45 ng/mL increase in UT-II concentration (B=36.45; SE=17.34; 95%CI 1.95–70.95; β=0.23; t=2.10; p=0.039). The explanatory power of the model was low (R^2^=0.05), with PAPP-A accounting for 5% of the variance in UT-II. According to the intercept, when PAPP-A is assumed to be zero, the UT-II level is estimated at 282.43 ng/mL (Table 3). The assumptions of the linear regression model were evaluated through residual diagnostics, including residual-versus-fitted plots, normal Q-Q plots, and histogram inspection. No substantial deviations from normality or homoscedasticity were observed.

**Table 3:** Simple linear regression analysis of the relationship between participants’ pregnancy-associated plasma protein-A concentration and urotensin-II levels.

Variable	B	SE	95%CI	β	t	p
Intercept	282.43	69.43	144.32–420.54	–	4.07	**<0.001**
PAPP-A concentration (mIU/mL)	36.45	17.34	1.95–70.95	0.23	2.10	0.039

B: unstandardized regression coefficient, β: standardized regression coefficient, SE: standard error; PAPP-A: pregnancy-associated plasma protein-A; CI: confidence interval; mIU/mL: milli-international unit per milliliter, t-statistic indicates whether the coefficient differs from zero, and p<0.05. It was considered statistically significant. Bold text indicates statistically significant values (p<0.05).

## DISCUSSION

This study was conducted to investigate the relationship between low PAPP-A levels and UT-II concentrations in pregnant women undergoing first-trimester combined screening. UT-II levels were found to be similar between the case and control groups (Table 1). Additionally, correlation analyses were performed comparing participants’ UT-II levels with biochemical and ultrasonographic parameters. A significant positive relationship was observed between UT-II levels and both PAPP-A concentration and PAPP-A MoM values (Table 2). This relationship was further examined using simple linear regression analysis. Although statistically significant positive correlations were observed between serum UT-II levels and both PAPP-A concentration and PAPP-A MoM values, the corresponding coefficients of determination (R^2^≈0.05) indicate that UT-II explains only a small proportion (~5%) of the variability in PAPP-A measurements (Table 3). Therefore, these associations should be interpreted as biologically suggestive rather than clinically predictive. Previous studies have demonstrated that low PAPP-A levels constitute a significant risk factor for adverse obstetric and perinatal outcomes^4,12,13^. Papamichail et al., in a cohort study, demonstrated that low PAPP-A levels measured in the first trimester are associated with an increased risk of preeclampsia, gestational hypertension, and FGR^14^. A study conducted in early gestation reported that low PAPP-A levels are associated with hypertensive pregnancy disorders, FGR, preterm birth, and early pregnancy loss^15^. Although low PAPP-A levels in the first trimester are associated with adverse pregnancy outcomes, it has been reported that PAPP-A alone has limited predictive value^13^. In this context, it is recommended that PAPP-A be integrated into multi-risk assessment approaches rather than being evaluated as a single marker in clinical practice^16,17^.

The influence of UT-II on biological pathways associated with endothelial dysfunction, along with its potent vasoconstrictive effect, suggests that this molecule may be involved in vascular pathophysiology during pregnancy. Some studies have demonstrated elevated placental UT-II levels, particularly in cases of preeclampsia^7,10,18^. It has been reported that in pregnant women diagnosed with severe preeclampsia, placental UT-II expression positively correlates with molecules associated with pyroptosis, an inflammatory cell death mechanism^10^. It has been reported that UT-II in the placentas of patients with preeclampsia correlates with markers of autophagy^7^. In the study by He et al., it was found that blood UT-II levels were higher in late-gestation pregnant women with hypertensive disorders compared to healthy pregnancies, whereas no significant differences were observed in urinary UT-II levels. The same study also demonstrated that in cases diagnosed with severe preeclampsia, UT-II levels were associated with endoplasmic reticulum stress markers, which were localized in the cytoplasm of placental trophoblastic cells^19^. These findings may indicate a potential role for UT-II in the regulation of placental stress responses. It has been suggested that elevated serum UT-II levels in pregnant women with preeclampsia may be associated with the severity or progression of the disease^20^. UT-II levels have been identified as an independent determinant of blood pressure in preeclampsia cases; in addition, UT-II protein expression is increased in the placentas of women diagnosed with severe preeclampsia, and this increase positively correlates with systolic blood pressure and urinary protein levels^21^. The involvement of UT-II in pregnancy extends beyond hypertensive disorders. In a study by Alaca et al., the relationship between UT-II levels measured in the placenta, umbilical cord, and blood samples and FGR was examined; while elevated UT-II levels were observed in tissue samples from pregnancies diagnosed with FGR, no increase was detected in serum samples^18^. In a study by Celik at al., no significant difference was found in terms of serum oxidant/antioxidant system parameters and U-II levels between the control group and the pregnancy group complicated with FGR^22^. When considered in the context of metabolic pregnancy complications, previous studies have reported elevated maternal plasma UT-II levels in women with gestational diabetes mellitus (GDM)^23^. Calan et al. reported that elevated UT-II levels were associated with insulin resistance among pregnant women with GDM^24^. Another study reported a significant decline in UT-II concentrations following delivery in healthy pregnant women without GDM; however, no significant change in postpartum UT-II levels was observed in women with GDM^25^. In a study of women with preeclampsia, serum UT-II levels demonstrated a significant positive correlation with HOMA-IR, a marker of insulin resistance^26^. These findings suggest that UT-II may be involved not only in vascular regulation but also in pathways related to metabolic stress and insulin resistance. Considering these studies on UT-II, it appears that UT-II levels are primarily associated with placental processes, and measurements have generally been conducted after adverse pregnancy complications have developed and during later gestational stages.

Evidence regarding UT-II levels in early pregnancy and their association with PAPP-A levels remains limited. The hypothesis of this study was that UT-II levels would be higher in the case group with low PAPP-A levels compared to the control group. However, no significant difference in UT-II was observed between the case and control groups. Nevertheless, analyses revealed a positive correlation between UT-II and PAPP-A levels. Based on these findings, it is suggested that UT-II may exert effects at the placental level, while the reflection of this activity in serum levels may be limited during early gestation.

This study may contribute to filling an important gap in the literature regarding the relationship between UT-II and PAPP-A levels in early pregnancy. However, the magnitude of the observed associations did not reach a level that would support the use of UT-II as a stand-alone clinical biomarker. Furthermore, the clinical interpretation of maternal serum UT-II concentrations remains challenging, as reference ranges and clinically validated threshold values for early pregnancy have not yet been established. Existing studies have primarily compared UT-II levels between specific pregnancy complications and control groups rather than defining gestational age–specific reference intervals or clinically relevant cut-off values. Therefore, although statistically significant, the observed differences should be interpreted cautiously with regard to their direct clinical applicability.

On the other hand, alternative explanations for the observed association should also be considered. First, residual confounding by unmeasured maternal, genetic, inflammatory, or environmental factors may have influenced both PAPP-A and UT-II levels. In addition to altered peptide signaling (PAPP A, UT-II), inherited variants affecting the renin–angiotensin system and fibrinolysis (e.g., ACE I/D and PAI 1 4G/5G) may modulate maternal–placental vascular responses and risk of adverse outcomes, suggesting that genetic predisposition could confound or interact with the biomarkers studied^27,28,29^.

Second, because both biomarkers are closely linked to placental physiology, the observed correlation may reflect a common placental origin rather than a direct biological interaction between the two molecules. According to studies, alterations in placental development, trophoblast function, or uteroplacental vascular adaptation could potentially affect both PAPP-A production and UT-II expression^7,30^.

Finally, although the ELISA assay used in this study has been reported by the manufacturer to have high specificity, the possibility of analytical variability or limited cross-reactivity inherent to immunoassay-based measurements cannot be completely excluded^31^. Therefore, the observed association should be interpreted with caution until confirmed by larger studies using complementary analytical approaches.

### Limitations and strengths

This case-control study is limited to 84 pregnant women who met the sample selection criteria at the hospital where the research was conducted. The lack of longitudinal data on pregnancy outcomes, such as preeclampsia, FGR, and preterm birth, limited the evaluation of the prognostic value of UT-II and low PAPP-A levels. Further prospective longitudinal studies are warranted to investigate their predictive significance for adverse obstetric outcomes. Moreover, future studies should consider genotyping for coagulation and vascular polymorphisms. The strengths of the study include the evaluation of UT-II during early pregnancy and the consideration of this molecule within the same pathophysiological framework as PAPP-A.

## CONCLUSION

In conclusion, this study found no association between low PAPP-A levels and UT-II concentrations in the case and control groups during early pregnancy. However, a significant positive relationship between UT-II levels and both PAPP-A concentration and PAPP-A MoM value was observed. UT-II may be involved in placental function during early gestation. Given that this study was conducted at a single center with a limited sample size, further research in multicenter studies with larger cohorts is recommended to determine the clinical relevance of this molecule.

## Data Availability

The datasets generated and/or analyzed during the current study are available from the corresponding author upon reasonable request.
